# Caregiver Burden in Head and Neck Cancer: The Combined Impact of Clinical Complexity and Psychological Distress

**DOI:** 10.3390/jcm15103713

**Published:** 2026-05-12

**Authors:** Yasar Kemal Duymaz, Seyma Akgun Bostanci, Huseyin Cubuk, Yesim Esen Yigit Kocer, Gamze Oruc Akcay, Ayse Asli Sahin Yılmaz

**Affiliations:** 1 Department of Otolaryngology, Sultan Abdulhamid Han Research and Training Hospital, 34668 Istanbul, Turkey; 2 Department of Otolaryngology, Umraniye Research and Training Hospital, 34764 Istanbul, Turkey; symakgn@gmail.com (S.A.B.); drhuseyincubuk@gmail.com (H.C.); dryesimesenyigit@gmail.com (Y.E.Y.K.); 3 Department of Psychiatry, Private Clinic, 34660 Istanbul, Turkey; gamzeakcay1@hotmail.com; 4 Department of Otolaryngology, Kartal Dr. Lutfi Kirdar Research and Training Hospital, 34865 Istanbul, Turkey; aslisahin@hotmail.com

**Keywords:** head and neck cancer, caregiver burden, psychological distress, depression, anxiety, quality of life

## Abstract

**Background/Objectives**: Caregiver burden represents a critical yet under-integrated dimension of clinical care in head and neck cancer, where complex treatments and functional impairments impose substantial demands on informal caregivers. This study aimed to evaluate caregiver burden and to identify its clinical and psychological correlates among caregivers of patients with head and neck cancer. **Methods**: This prospective cross-sectional study included 132 caregivers. Caregiver burden was assessed using the Zarit Burden Interview, and psychological distress was evaluated using the Hamilton Depression (HAM-D) and Anxiety (HAM-A) Rating Scales. Clinical variables, including tracheostomy status, radiotherapy, disease stage, chemotherapy, reconstructive surgery, and disease recurrence, were analyzed. Univariate and multivariate analyses were performed to identify factors associated with caregiver burden. Statistical significance was defined as *p* < 0.05. **Results**: Caregiver burden was highly prevalent, with a substantial proportion of participants experiencing moderate to severe levels. In the univariate analyses, caregiver burden was significantly higher among caregivers of patients who underwent tracheostomy (*p* = 0.003), radiotherapy (*p* < 0.001), chemotherapy (*p* < 0.001), reconstructive surgery (*p* = 0.024), and those with advanced-stage disease (*p* < 0.001). Higher levels of depression and anxiety were significantly associated with increased caregiver burden (both *p* < 0.001) in the univariate analyses. In the adjusted analysis, anxiety and cohabitation status remained independently associated with caregiver burden, whereas disease stage, tracheostomy, radiotherapy, chemotherapy, reconstructive surgery, disease recurrence, and depression did not retain statistical significance. Educational level, professional caregiving support, and relationship to the patient were also not significantly associated with caregiver burden. **Conclusions**: Caregiver burden in head and neck cancer is primarily associated with caregiver psychological status and certain social characteristics, while clinical factors play a less prominent role after adjustment. These findings suggest the need for systematic identification of high-risk caregivers based on psychological vulnerability and caregiving demands. Integrating caregiver-focused assessment and targeted psychosocial interventions into multidisciplinary care could improve caregiver well-being and patient-related outcomes.

## 1. Introduction

Head and neck cancers comprise a heterogeneous group of malignancies arising from the upper aerodigestive tract and represent a significant global health burden due to their high morbidity and mortality. These cancers profoundly affect essential functions such as speech, swallowing, and breathing, leading to substantial impairments in patients’ quality of life. Despite advances in surgical and oncological treatments, patients continue to experience complex clinical courses associated with considerable functional and psychosocial challenges [1,2].

The complex and prolonged treatment process of head and neck cancer not only affects patients but also places a substantial burden on family members who assume caregiving responsibilities. Informal caregivers play a critical role in supporting patients throughout diagnosis, treatment, and recovery, often undertaking physically demanding and emotionally challenging tasks. The concept of caregiver burden encompasses the physical, psychological, social, and economic strain experienced by individuals providing care. In head and neck cancer, caregiving is particularly demanding due to disease-specific challenges such as communication difficulties, nutritional problems, and visible disfigurement, all of which may significantly affect caregivers’ well-being. Caregivers have been shown to experience disruptions to their daily routines, emotional distress, and social limitations throughout the caregiving process [3,4,5].

The caregiver burden in head and neck cancer is influenced not only by disease-related clinical factors, but also by the psychological status of the caregiver. Uncertainty regarding disease progression and increasing caregiving responsibilities may contribute to significant psychological distress. Previous studies have consistently demonstrated that higher levels of depression and anxiety are associated with increased caregiver burden in cancer populations [6]. In addition, unmet supportive care needs and increasing care demands have been identified as important contributors to caregiver burden [7,8]. Together, these findings suggest a dynamic interaction between clinical burden and psychological distress in shaping caregiver burden.

However, despite increasing recognition of caregiver burden in oncology, the combined impact of disease-specific clinical factors and caregiver psychological status has not been sufficiently clarified in head and neck cancer populations. In particular, the contribution of treatment-related factors such as tracheostomy, radiotherapy, and disease stage to caregiver burden, alongside psychological distress, remains underexplored.

Therefore, the present study aimed to evaluate caregiver burden in caregivers of patients with head and neck cancer and to investigate its association with both clinical characteristics and psychological factors. By identifying key determinants of caregiver burden, this study seeks to provide clinically relevant insights to support risk stratification and the integration of caregiver-focused assessment into multidisciplinary cancer care.

## 2. Materials and Methods

This study was designed as a prospective, single-center, analytical, cross-sectional study to evaluate caregiver burden and psychological status among caregivers of patients diagnosed with head and neck cancer. The study was conducted from November 2021 to November 2022 at the Otorhinolaryngology Department of the University of Health Sciences’ Ümraniye Training and Research Hospital. Since the study used a consecutive convenience sample within a predefined recruitment window, a formal a priori sample size calculation was not performed. To assess the adequacy of the achieved sample, a post hoc power analysis was conducted.

The participants included caregivers of patients diagnosed with head and neck cancer who were aged 18 years or older. Individuals with a history of psychiatric disorders, those using antidepressant or antipsychotic medications, those who were unable to speak Turkish, and those who declined to participate were excluded from the study. The sample size was determined by including all eligible caregivers during the study period.

The demographic and clinical characteristics of both patients and caregivers, including age, gender, education level, socioeconomic status, comorbidities, disease stage, treatment modalities, and tumor recurrence, were recorded.

Data were collected through face-to-face interviews with caregivers during routine outpatient visits. Patients were in different phases of the disease trajectory, including post-surgical follow-up, ongoing radiotherapy, and surveillance. Caregivers were also assessed across these clinical phases. In addition to a sociodemographic data form, standardized scales were administered to evaluate caregiver burden, depression, and anxiety levels. Caregiver burden was assessed using the Zarit Burden Interview, a 22-item survey that uses a Likert-type scale to score responses; it was developed by Zarit et al. to evaluate the physical, psychological, social, and economic burden experienced by caregivers [9]. The validity and reliability of the Turkish version of this questionnaire have been established in previous studies [10].

Depression levels were evaluated using the Hamilton Depression Rating Scale (HAM-D), a 17-item clinician-administered scale developed by Hamilton [11]. The validity and reliability of the Turkish version of this scale were previously confirmed [12]. Anxiety levels were assessed using the Hamilton Anxiety Rating Scale (HAM-A), a 14-item scale also developed by Hamilton [13]; the validity and reliability of the Turkish version of this scale have also been confirmed [14]. Both scales were administered by a psychiatrist through face-to-face clinical interviews using a standardized interview procedure.

Statistical analyses were performed using SPSS version 28.0 (IBM Corp., Armonk, NY, USA). Continuous variables are expressed as means ± SDs, and categorical variables are presented as frequencies and percentages. Data normality was assessed using the Shapiro–Wilk test, which is generally preferred over the Kolmogorov–Smirnov test for sample sizes below 2000 due to its higher sensitivity in detecting deviations from normality. As distributions were found to be non-normal, non-parametric tests were used for group comparisons. Continuous variables were compared using the Kruskal–Wallis test (ε^2^), and categorical variables were analyzed using Chi-square or Fisher’s exact tests (Cramér’s V). Effect sizes were interpreted as small, medium, and large using thresholds of 0.01, 0.06, and 0.14 for ε^2^, and ≤0.10, 0.10–0.30, and ≥0.30 for Cramér’s V, respectively. To account for multiple comparisons in univariate analyses, Bonferroni correction was applied. Ordinal logistic regression was conducted to identify independent predictors of caregiver burden, with model fit assessed using AIC, BIC, and McFadden’s R^2^. Odds ratios (ORs) and 95% confidence intervals (CIs) are reported for all predictors. To confirm the robustness of our primary finding regarding anxiety, a post hoc power analysis was conducted. Assuming an R^2^ of 0.30 for the other predictors to account for potential multicollinearity and based on the observed odds ratio (OR = 3.28), an alpha error probability of 0.05, and a sample size of 132, the achieved statistical power was 0.988. Except where adjusted by the Bonferroni method, *p* < 0.05 was considered statistically significant.

Ethical approval for the study was obtained from the Clinical Research Ethics Committee of the University of Health Sciences’ Ümraniye Training and Research Hospital (decision No. B.10.1.TKH.4.34.H.GP.0.01/287, dated 30 September 2021). The study was conducted in accordance with the principles of the Declaration of Helsinki.

## 3. Results

The baseline characteristics of the participants are presented in Table 1. A total of 132 caregivers were included in the study. The mean age was 51.0 ± 11.6 years (range: 21–77), and 78.8% were female. Most caregivers lived in the same household as the patient (86.4%) and were primarily spouses (59.8%) or children (30.3%).

Regarding clinical characteristics of the patients, the tumors were most commonly located in the larynx (50.8%) and oral cavity (36.4%). Disease stage was predominantly stage II (34.1%) and stage III (31.1%). In terms of treatment and clinical course, 46.2% of the patients had received radiotherapy, 20.5% had received chemotherapy, 23.5% had undergone reconstructive surgery, and tumor recurrence was observed in 12.9% of patients.

Moderate to severe caregiver burden was observed in the majority of participants, with 41.7% classified as moderate and 22.0% as severe. In terms of psychological status, moderate to severe depression was present in 53.8% of caregivers, while moderate to severe anxiety was observed in 58.3%.

When comparisons were made according to caregiver burden levels, no significant differences were observed between the groups in terms of age (*p* = 0.572) and gender (*p* = 0.934). Caregiver burden was significantly higher among caregivers of patients that received a tracheostomy (*p* = 0.003) and those receiving radiotherapy (*p* < 0.001). Caregiver burden was also significantly higher among patients receiving chemotherapy (*p* < 0.001) and those who had undergone reconstructive surgery (*p* = 0.024). In addition, caregiver burden was significantly associated with advanced disease stages, with higher levels observed in stage III–IV patients (*p* < 0.001) (Table 2). No significant association was found between caregiver burden and tumor localization (*p* > 0.05) or tumor recurrence (*p* = 0.470).

No significant differences in caregiver burden were observed according to education level (*p* = 0.887), cohabitation status (*p* = 0.697), professional caregiving support (*p* = 0.957), or relationship to the patient (*p* = 0.506). In contrast, caregiver burden increased significantly with higher levels of depression (*p* < 0.001) and anxiety (*p* < 0.001) (Table 3).

An ordinal logistic regression analysis was performed to identify independent predictors of caregiver burden. The combined model that included both anxiety and depression provided the best fit (Table 4). In the adjusted analysis, cohabitation status (OR = 0.148, 95% CI: 0.025–0.783, *p* = 0.027) and anxiety (OR = 3.281, 95% CI: 1.325–8.67, *p* = 0.012) were identified as significant independent predictors of caregiver burden. Tracheostomy showed a trend toward statistical significance (*p* = 0.057). In contrast, disease stage, radiotherapy, chemotherapy, reconstructive surgery, tumor recurrence, depression, and the other sociodemographic variables did not retain statistical significance after adjustment (Table 5).

## 4. Discussion

In this study, the caregiver burden among caregivers of patients with head and neck cancer was predominantly moderate to high. In the univariate analyses, caregiver burden was associated with several clinical and psychological variables, including tracheostomy, radiotherapy, advanced disease stage, chemotherapy, reconstructive surgery, depression, and anxiety. However, in the adjusted analysis, cohabitation status and anxiety remained independently associated with caregiver burden, while disease stage, radiotherapy, tracheostomy, depression, chemotherapy, reconstructive surgery, and tumor recurrence did not retain statistical significance. These findings suggest that the caregiver burden in this population is associated with psychological factors and certain social characteristics, while the role of clinical factors appears to be less pronounced after adjustment.

These findings are consistent with the existing literature, which has repeatedly documented substantial caregiver burden and elevated psychological distress among caregivers of patients with head and neck cancer [15,16,17]. The complex and prolonged care process, characterized by functional impairments such as dysphagia, speech difficulties, and disfigurement, places substantial physical and emotional demands on caregivers. Taken together, these results suggest that caregiver burden in head and neck cancer is not only prevalent, but also clinically relevant, warranting systematic attention within routine cancer care.

In our study, caregiver burden was significantly associated with the presence of a tracheostomy, receipt of radiotherapy, and disease stage in the univariate analyses. Similarly, chemotherapy and reconstructive surgery were also associated with higher caregiver burden in the univariate analyses. These findings suggest that treatment-related clinical complexity may be associated with caregiver burden in head and neck cancer. Although tracheostomy was associated with caregiver burden in the univariate analyses, this association demonstrated a trend but did not reach statistical significance in the adjusted model. Tracheostomy care requires continuous monitoring, secretion management, and technical skills, which may increase caregiving demands and be associated with higher levels of caregiver stress. Previous studies have also suggested that complex care tasks, including tracheostomy management and enteral feeding, may be associated with increased caregiver burden and psychological distress [7,18]. Taken together, tracheostomy may be linked to increased caregiving demands; however, it does not appear to be an independent predictor of caregiver burden when potential confounding factors are taken into account. Similarly, radiotherapy and chemotherapy are often accompanied by a high symptom burden, including mucositis, dysphagia, fatigue, and general functional decline, which may increase caregiving demands. In addition, reconstructive surgery may further increase caregiving complexity due to postoperative care needs and functional limitations. These treatment-related factors are frequently observed in patients with more advanced disease, which may partly explain their associations with caregiver burden in the univariate analyses. Consistent with this, previous studies have reported that caregivers of patients with advanced disease experience higher levels of both physical and psychological burdens [19,20]. However, in the multivariate analysis, the associations between disease stage, radiotherapy, chemotherapy, and reconstructive surgery and caregiver burden did not retain statistical significance. This suggests that the effects observed in the univariate analyses may be influenced by overlapping clinical characteristics and the overall burden of the caregiving process.

Caregiver burden was significantly associated with both depression and anxiety levels in the univariate analyses, suggesting that psychological distress represents an important dimension of caregiver burden. However, in the multivariate analysis, anxiety remained independently associated with caregiver burden, whereas depression did not retain statistical significance. This pattern may be related to the substantial overlap between these constructs. Previous studies have reported high levels of psychological distress among caregivers of patients with head and neck cancer, and both depression and anxiety have been associated with increased caregiver burden. In addition, caregiving-related challenges such as a high symptom burden, communication difficulties, and functional impairments have been linked to psychological distress [21,22,23]. Within this context, caregiving demands may be more closely aligned with anxiety-related experiences, including uncertainty, heightened vigilance, and emotional strain. When evaluated together, anxiety remained independently associated with caregiver burden. This pattern is consistent with the view that caregiving demands and psychological distress coexist and may reinforce one another. Accordingly, caregiver burden can be conceptualized as a multidimensional construct primarily shaped by psychological factors. Routine psychological assessment and timely supportive interventions for caregivers may therefore represent important components of comprehensive care for head and neck cancer patients.

The multivariate analysis provides a more comprehensive assessment by identifying variables that remain independently associated with caregiver burden after adjustment. In this model, cohabitation status and anxiety were independently associated with caregiver burden, suggesting that living arrangements may be related to caregiving responsibilities, although the underlying mechanisms remain unclear. Variables that were significant in the univariate analyses, including disease stage, radiotherapy, tracheostomy, chemotherapy, and reconstructive surgery, did not retain statistical significance after adjustment, which may reflect the influence of other clinical factors and the overall burden of the caregiving process rather than independent effects.

Most sociodemographic characteristics were not significantly associated with caregiver burden. Educational level, professional caregiving support, and relationship to the patient were not associated with caregiver burden, whereas cohabitation status was independently associated with caregiver burden in the multivariate analysis. The literature regarding the impact of sociodemographic factors on caregiver burden is heterogeneous. While some studies report associations with variables such as age, gender, and education, others have found no significant relationship [24,25]. These inconsistencies could be explained by differences in disease characteristics, caregiving demands, and sociocultural contexts. Taken together, caregiver burden in head and neck cancer may be more closely associated with caregiving process-related factors and psychological dimensions; certain social factors may also be relevant, but caregiver burden cannot be explained solely by sociodemographic characteristics [6].

The findings of this study suggest that caregiver burden in individuals providing care to patients with head and neck cancer may be primarily associated with the psychological status of the caregiver, particularly their anxiety, while clinical characteristics may play a less prominent role after adjustment. These results may be interpreted within a broader framework in which caregiver burden reflects a multidimensional construct shaped by overlapping psychological and caregiving-related demands rather than isolated variables. This perspective underscores the potential relevance of a holistic and multidisciplinary approach that incorporates caregiver-focused assessment into routine clinical practice. Accordingly, routine psychological evaluation and timely supportive interventions for caregivers could be included in comprehensive care practices for head and neck cancer patients.

This study has several limitations that should be considered when interpreting the findings. First, the single-center design may limit the generalizability of the results. Second, the cross-sectional nature of the study precludes causal inferences between variables. Third, the absence of an a priori sample size calculation represents a methodological limitation. In addition, the use of self-reported measures may have introduced bias, including recall and social desirability biases. Furthermore, the dynamic nature of caregiving and changes in caregiver burden over time could not be assessed. Caregivers were assessed across different phases of the disease trajectory since caregiver burden reflects a heterogeneous clinical context rather than a single, uniform time point. Relevant factors such as psychosocial support, coping strategies, and social support systems were not examined in detail and may have influenced the findings. Another limitation of this study is the exclusion of caregivers with a history of psychiatric disorders or those receiving antidepressant or antipsychotic treatment, which may have introduced selection bias by potentially underrepresenting individuals at higher risk of psychological distress and caregiver burden. This may have led to an underestimation of the true burden observed in real-world clinical settings. Future studies with larger sample sizes, multicenter designs, and longitudinal approaches are needed to provide a more comprehensive understanding of caregiver burden in this population.

## 5. Conclusions

In conclusion, caregivers of patients with head and neck cancer experience a substantial caregiver burden, which appears to be associated primarily with the psychological status of the caregiver, while clinical factors play a less prominent role after adjustment. In the adjusted analysis, anxiety and cohabitation status were independently associated with caregiver burden, whereas clinical variables did not retain statistical significance. These findings suggest the importance of recognizing caregivers as an integral part of the cancer care process. Routine assessment of caregiver burden and psychological well-being, along with the provision of appropriate supportive interventions, could be considered in clinical practice. Adopting a multidisciplinary and holistic approach that addresses both patient and caregiver needs could improve overall care outcomes.

## Figures and Tables

**Table 1:** Characteristics of caregivers and clinical features of patients.

	Mean ± SD/*n* %		
Age	51.0 ± 11.6		
Gender	Female	104	78.8%
	Male	28	21.2%
Tracheostomy	(−)	101	76.5%
	(+)	31	23.5%
Localization	Larynx	67	50.8%
	Oral Cavity	48	36.4%
	Paranasal Sinus	5	3.8%
	Salivary Gland	12	9.1%
Radiotherapy	(−)	71	53.8%
	(+)	61	46.2%
Chemotherapy	(−)	105	79.5%
	(+)	27	20.5%
Reconstructive Surgery	(−)	101	76.5%
	(+)	31	23.5%
Recurrence	(−)	115	87.1%
	(+)	17	12.9%
Stage	I	25	18.9%
	II	45	34.1%
	III	41	31.1%
	IV	21	15.9%
Depression	Minimal	37	28.0%
	Mild	24	18.2%
	Moderate	44	33.3%
	Severe	27	20.5%
Anxiety	Minimal	36	27.3%
	Mild	19	14.4%
	Moderate	47	35.6%
	Severe	30	22.7%
Education Level	Elementary	91	68.9%
	High School	34	25.8%
	University	7	5.3%
Cohabitation	(−)	18	13.6%
	(+)	114	86.4%
Professional Caregiver	(−)	120	90.9%
	(+)	12	9.1%
Relationship	Child	40	30.3%
	Spouse	79	59.8%
	Other	13	9.8%
Caregiver Burden (Zarit Burden Interview Score)	Mild	48	36.4%
	Moderate	55	41.7%
	Severe	29	22.0%

**Table 2:** Comparison of demographic and clinical variables according to caregiver burden levels.

	Mild Caregiver Burden	Moderate Caregiver Burden	Severe Caregiver Burden	*p*	Effect Size	Test
Age (mean ± SD)	52.0 ± 12.4	50.1 ± 11.8	51.0 ± 10.0	0.572	ε^2^ = 0.009	K
Gender				0.934	V = 0.032	X^2^
Female	37 (77.1%)	44 (80.0%)	23 (79.3%)			
Male	11 (22.9%)	11 (20.0%)	6 (20.7%)			
Tracheostomy				**0.003**	V = 0.298	X^2^
(−)	44 (91.7%)	40 (72.7%)	17 (58.6%)			
(+)	4 (8.3%)	15 (27.3%)	12 (41.4%)			
Localization				0.418	V = 0.164	X^2^
Larynx	21 (43.8%)	29 (52.7%)	17 (58.6%)			
Oral cavity	17 (35.4%)	22 (40.0%)	9 (31.0%)			
Paranasal sinus	3 (6.3%)	1 (1.8%)	1 (3.4%)			
Salivary gland	7 (14.6%)	3 (5.5%)	2 (6.9%)			
Radiotherapy				**<0.001**	V = 0.645	X^2^
(−)	44 (91.7%)	25 (45.5%)	2 (6.9%)			
(+)	4 (8.3%)	30 (54.5%)	27 (93.1%)			
Stage				**<0.001**	V = 0.519	X^2^
I	21 (43.8%)	4 (7.3%)	0 (0.0%)			
II	22 (45.8%)	21 (38.2%)	2 (6.9%)			
III	5 (10.4%)	23 (41.8%)	13 (44.8%)			
IV	0 (0.0%)	7 (12.7%)	14 (48.3%)			
Chemotherapy				**<0.001**	V = 0.456	X^2^
(−)	47 (97.9%)	44 (80%)	14 (48.3%)			
(+)	1 (2.1%)	11 (20%)	15 (51.7%)			
Reconstructive surgery				**0.024**	V = 0.238	X^2^
(−)	43 (89.6%)	39 (70.9%)	19 (65.5%)			
(+)	5 (10.4%)	16 (29.1%)	10 (34.5%)			
Recurrence				0.470	V = 0.107	X^2^
(−)	44 (91.7%)	47 (85.5%)	24 (82.8%)			
(+)	4 (8.3%)	8 (14.5%)	5 (17.2%)			

Notes: X^2^ = Chi-square test; K = Kruskal–Wallis test; V = Cramér’s V; ε^2^ = epsilon squared. Bold *p*-values indicate significance at α = 0.05. Effect size interpretation for Cramér’s V: small (≤0.10), medium (0.10–0.30), large (≥0.30).

**Table 3:** Association between sociodemographic and psychological variables and caregiver burden level.

Variable		Mild Caregiver Burden, *n* (%)	Moderate Caregiver Burden, *n* (%)	Severe Caregiver Burden, *n* (%)	*p*
Education Level	Elementary	34 (70.8)	38 (69.1)	19 (65.5)	0.887 χ^2^
	High School	12 (25.0)	14 (25.5)	8 (27.6)	
	University	2 (4.2)	3 (5.5)	2 (6.9)	
Cohabitation	No	8 (16.7)	6 (10.9)	4 (13.8)	0.697 χ^2^
	Yes	40 (83.3)	49 (89.1)	25 (86.2)	
Professional caregiver	No	44 (91.7)	50 (90.9)	26 (89.7)	0.957 χ^2^
	Yes	4 (8.3)	5 (9.1)	3 (10.3)	
Relationship	Child	18 (37.5)	16 (29.1)	6 (20.7)	0.50 6χ^2^
	Spouse	25 (52.1)	33 (60.0)	21 (72.4)	
	Other	5 (10.4)	6 (10.9)	2 (6.9)	
Depression	Minimal	35 (72.9)	2 (3.6)	0 (0.0)	<0.001 χ^2^
	Mild	8 ^1,2^ (16.7)	14 ^2^ (25.5)	2 (6.9)	
	Moderate	3 (6.3)	29 (52.7)	12 (41.4)	
	Severe	2 (4.2)	10 (18.2)	15 (51.7)	
Anxiety	Minimal	35 (72.9)	1 (1.8)	0 (0.0)	<0.001 χ^2^
	Mild	6 ^1,2^ (12.5)	12 ^2^ (21.8)	1 (3.4)	
	Moderate	6 (12.5)	30 (54.5)	11 (37.9)	
	Severe	1 (2.1)	12 (21.8)	17 (58.6)	

χ ^2^ Chi-square test. ^1^ Difference compared to moderate caregiver burden group (*p* < 0.05). ^2^ Difference compared with severe caregiver burden group (*p* < 0.05).

**Table 4:** Fit of ordinal logistic regression models.

	Deviance	AIC	BIC	R^2^McF	*p*
Model A (Anxiety Only)	156	192	244	0.446	<0.001
Model B (Depression Only)	160	196	248	0.431	<0.001
Model C (Both)	**153**	**191**	246	**0.455**	<0.001

Note. All models were adjusted for age, sex, tracheostomy, radiotherapy, localization, stage, cohabitation, education level, professional caregiver, and relationship to patient. Bold values indicate the best fit. *n* = 132.

**Table 5:** Ordinal logistic regression coefficients for caregiver burden (Model C).

						95% CI	
Predictor	Est.	SE	*Z*	*p*	OR	Lower	Upper
Age	0.027	0.020	1.331	0.183	1.028	0.987	1.071
Localization (ref: larynx)							
Oral cavity	0.553	0.817	0.677	0.498	1.739	0.346	8.76
Oropharynx	−0.157	0.920	−0.171	0.864	0.854	0.129	4.93
Paranasal sinus	−1.180	1.464	−0.806	0.420	0.307	0.013	5.32
Salivary gland	−0.167	0.861	−0.194	0.846	0.846	0.150	4.56
RT (ref: no)							
Yes	0.229	0.984	0.232	0.816	1.258	0.177	8.69
Sex (ref: male)							
Female	0.108	0.535	0.203	0.839	1.115	0.390	3.230
Cohabitation (ref: no)							
Yes	−1.907	0.864	−2.205	**0.027**	0.148	0.025	0.783
Tracheostomy (ref: no)							
Yes	−1.273	0.666	−1.904	0.057	0.280	0.071	1.007
Stage	1.093	0.659	1.657	0.097	2.984	0.841	11.42
Education (ref: elementary)							
High school	−0.526	0.527	−0.997	0.319	0.591	0.206	1.65
University	0.663	1.015	0.653	0.513	1.942	0.269	16.12
Prof. caregiver (ref: yes)							
No	−0.020	0.730	−0.027	0.978	0.980	0.235	4.30
Relationship (ref: other)							
Spouse	0.606	0.795	0.762	0.446	1.835	0.393	9.02
Child	−1.493	0.902	−1.655	0.098	0.225	0.036	1.30
Chemotherapy (ref: no)							
Yes	0.277	0.810	0.341	0.732	1.319	0.265	6.48
Reconstructive surgery (ref: no)							
Yes	0.286	0.803	0.356	0.721	1.332	0.278	6.65
Recurrence (ref: no)							
Yes	0.011	0.657	0.017	0.986	1.012	0.270	3.661
Depression	0.744	0.479	1.554	0.120	2.106	0.820	5.43
Anxiety	1.188	0.475	2.500	**0.012**	3.281	1.325	8.67

Notes: OR = odds ratio; CI = confidence interval; ref = reference category. Dependent variable: Zarit Burden Interview score (mild, moderate, or severe). Bold *p*-values indicate significance at α = 0.05. *n* = 132.

## Data Availability

The datasets presented in this study are not publicly available due to privacy and ethical restrictions. Requests to access the datasets should be directed to the corresponding author.
